# Physio-Biochemical Responses of Sweet Cherry Leaf to Natural Cold Conditions

**DOI:** 10.3390/plants11243507

**Published:** 2022-12-14

**Authors:** Matej Vosnjak, Helena Sircelj, Dominik Vodnik, Valentina Usenik

**Affiliations:** Department of Agronomy, Biotechnical Faculty, University of Ljubljana, Jamnikarjeva 101, SI-1000 Ljubljana, Slovenia

**Keywords:** cold stress, photosynthesis, pigments, *Prunus avium*, recovery

## Abstract

Trees of the sweet cherry cultivar ‘Grace Star’ (*Prunus avium* L.) were exposed to low temperatures without frost for two consecutive nights under natural conditions 36 d after flowering, to study the effects on the physiological properties and metabolic status of leaves. The response was studied by measuring chlorophyll fluorescence and gas exchange parameters and by analyzing chloroplast pigments (i) immediately after exposure, (ii) 24 h and (iii) 48 h later. The first exposure at 2.4 (±0.2) °C and a minimum of 0.8 °C elicited more changes than the second exposure at 4.9 (±0.3) °C and a minimum of 2.4 °C. After the first exposure, the maximum quantum yield of PS II (Fv/Fm), effective quantum efficiency of PS II, net photosynthesis (P_N_), stomatal conductance (*g_s_*), transpiration, and intercellular CO_2_ concentration were significantly lower, and after the second exposure, the content of chlorophyll *b*, total chlorophyll, *β*-carotene, and lutein were lower. The content of antheraxanthin and zeaxanthin was higher immediately after both exposures, and that of antheraxanthin was also higher 24 h later. Recovery took longer in trees that were exposed twice. Fv/Fm recovered within 48 h, but the de-epoxidation state of the xanthophyll cycle pool, P_N_, and *g_s_* did not reach the level of controls, indicating that the stress effect lasted several days which is probably sufficient to cause fruit drop and reduce yield.

## 1. Introduction

Sweet cherry (*Prunus avium* L.), an economically important fruit species, can be grown under a wide range of climatic conditions. However, climate change is causing earlier flowering. As a result, sweet cherry production is more threatened than in the past [1]. The most important climate factor limiting sweet cherry production is spring frost, but sweet cherry trees are also often exposed to low temperatures without frost (temperatures above zero but below the threshold of 4.5 °C) early in the growing season. However, comprehensive analyses of plant physiological responses to this type of stress are rare, although such low temperatures force plants to activate various physiological and biochemical processes [2,3].

Physiological limitations due to cold stress mainly result from a disturbed plant membrane system, especially protein/lipid ratio and fatty acid saturation [4,5]. As a result, various plant physiological processes such as stomatal conductance, leaf internal CO_2_ concentration, Rubisco activity, carbon reduction cycle, thylakoid electron transport, net photosynthesis [6,7,8], and chlorophyll fluorescence [9,10] are affected. The effects of low temperature on photosynthetic apparatus function have been studied in sensitive plants [9,10,11,12,13]. However, there are few studies investigating the effects of artificially induced cold stress on the response of temperate fruit species and none on the effects of cold stress under natural conditions. The inhibitory effect of temperatures of 3–5 °C on photosynthesis and photochemical efficiency of PS II has been reported, as well as the increase in antioxidant enzymes and the decrease in chlorophyll and carotenoid content in leaves [14,15,16,17,18].

The response of sweet cherry leaves to low temperatures was investigated in our previous study [19,20]. Here, cold stress was artificially induced by exposing potted trees to 4.7 or 2.2 °C for 1, 2 or 3 consecutive nights 3 or 7 weeks after flowering. Exposure of sweet cherry trees to 2.2 °C reduced photochemical efficiency, net photosynthesis, stomatal conductance, and transpiration. Two or three exposures increased leaf content of sugars, phenolics, and zeaxanthin. In addition, multiple exposures caused greater physiological disruption than a single exposure and prolonged recovery time. Trees exposed three times did not recover within 48 h after the last exposure, whereas trees exposed once or twice did. No differences were found in the response of the two cultivars [19,20].

The objective of the present study was to investigate the effects of exposure of 3-year-old sweet cherry trees to low temperatures under natural conditions on leaf physiological characteristics and metabolic status, and to determine whether exposure of sweet cherry trees to low temperatures under natural and artificial conditions produces the same response.

## 2. Results

### 2.1. Chlorophyll Fluorescence Parameters

**Figure 1:**
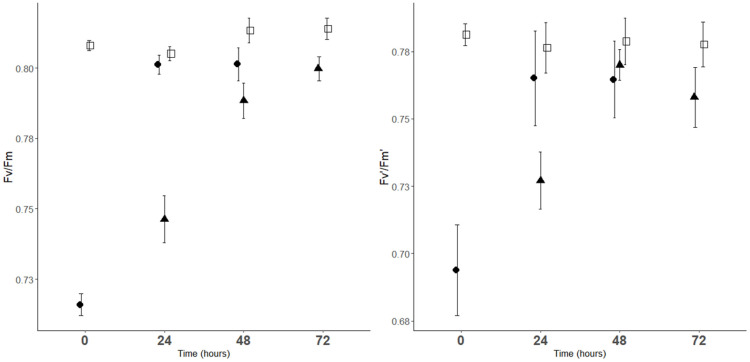
The maximum quantum efficiency of PS II (Fv/Fm) and the effective quantum efficiency of PS II (Fv’/Fm’) of sweet cherry leaf, at time 0, time 24, time 48 and time 72. ●, CT1 (one cold night exposure); ▲, CT2 (two cold night exposures) and □, C (control trees). Vertical bars represent ± SE of the mean (*n* = 6 or 9).

Analysis of variance showed significant effects of CT.time on Fv/Fm, Fv’/Fm’ and ETR (*p* < 0.001). CT1 and CT2 trees had a significantly lower average Fv/Fm and Fv’/Fm’ than the corresponding controls (Figure 1 and Table 1). Fv/Fm and Fv’/Fm’ of CT1 trees recovered within 24 h, whereas CT2 trees recovered more slowly, within 48 h, when they reached values similar to those of the control. CT2 trees had a significantly higher average ETR than the corresponding controls immediately after exposure (Figure 1 and Table 1).

### 2.2. Gas Exchange Parameters

**Figure 2:**
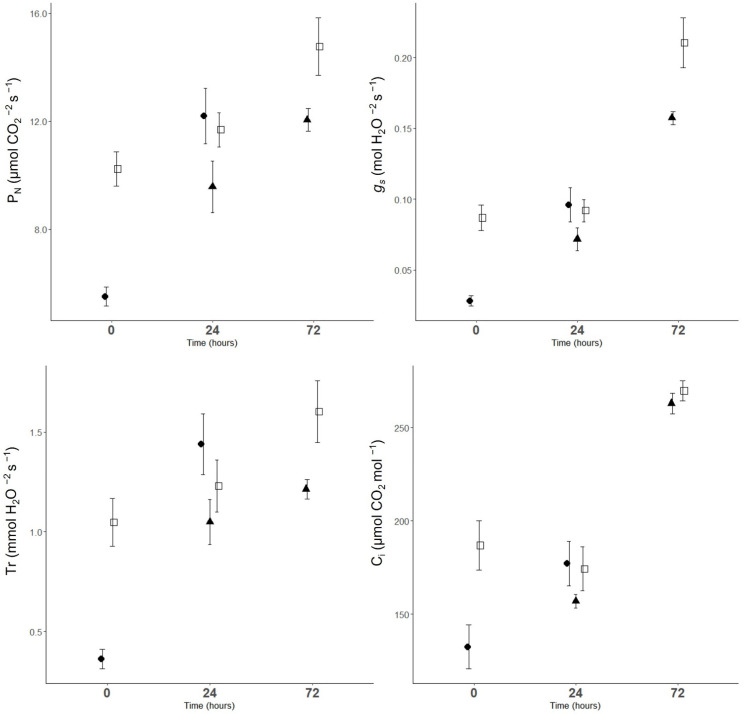
Net photosynthesis (P_N_), stomatal conductance (*g_s_*), transpiration (Tr) and intercellular leaf CO_2_ concentration (C*_i_*) of sweet cherry leaf, at time 0, time 24 and time 72. ●, CT1 (one cold night exposure); ▲, CT2 (two cold night exposures) and □, C (control trees). Vertical bars represent ± SE of the mean (*n* = 6 and 9).

CT.time significantly affected all measured gas exchange parameters (*p* < 0.001). After the first night of cold, leaves had significantly lower P_N_, *g_s_*, Tr and C*_i_* (*p* < 0.001) (Figure 2 and Table 1), while the second night of cold had no significant effect on gas exchange parameters compared to the corresponding controls. After the first cold night, leaves recovered within 24 h and reached similar values to the corresponding controls, but trees exposed twice did not recover even after 72 h.

### 2.3. Biochemical Parameters

The predominant carotenoid in sweet cherry leaves was lutein, which accounted for an average of 32% of the total carotenoids, followed by *β*-carotene (28%), violaxanthin (17%), neoxanthin (14%), antheraxanthin (8%) and zeaxanthin (1%). The major xanthophyll cycle pigment was violaxanthin (average 66% of xanthophyll), followed by antheraxanthin (30%) and zeaxanthin (3%). Chlorophyll *a* was the major chlorophyll pigment (averaging of 64% of total chlorophylls).

**Figure 3:**
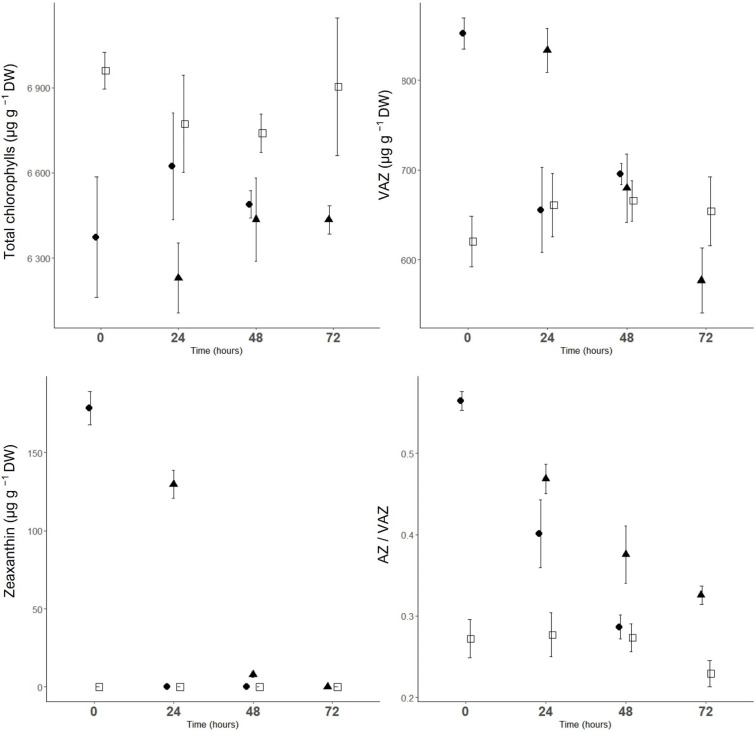
The content of total chlorophylls, VAZ (xanthophyll cycle pigments) and zeaxanthin and AZ/VAZ ratio (de-epoxidation state of xanthophyll cycle pool) in sweet cherry leaf, at time 0, time 24, time 48 and time 72. ●, CT1 (one cold night exposure); ▲, CT2 (two cold night exposures) and □, C (control trees). Vertical bars represent ± SE of the mean (*n* = 3; *n* = 3 and 6 for controls).

CT.time significantly affected the content of all individual chlorophylls and carotenoids (except chlorophyll *a* and neoxanthin), total chlorophylls, chlorophyll *a*/*b* ratio, and VAZ and AZ/VAZ (Figure 3 and Table 1). Further contrast analysis showed significant differences after the first and second cold night compared to the corresponding controls.

After the first and second cold nights, trees had significantly higher contents of antheraxanthin, zeaxanthin and VAZ, higher ratios of chlorophyll *a*/*b* and AZ/VAZ, lower contents of chlorophyll *b*, *β*-carotene, lutein, and total chlorophylls compared with controls (Figure 3 and Table 1). Of the VAZ, zeaxanthin and antheraxanthin were the most affected and increased, while violaxanthin decreased significantly only after the first cold night. VAZ increased by 38% and 26% and the sum of zeaxanthin and antheraxanthin increased by 186% and 113%, respectively, after the first and second cold night compared to controls.

The content of zeaxanthin, chlorophyll *b*, total chlorophyll, and VAZ reached values similar to those of the corresponding controls within 24 h after CT1 and CT2. The AZ/VAZ ratio of CT1 trees 48 h after exposure (time 48) was similar to the values of the corresponding controls, whereas it was significantly higher in CT2 trees (time 72). At this time point, the β-carotene content of the CT2 trees also remained significantly lower than that of the corresponding controls (Table 1).

## 3. Discussion

The physiological and biochemical response of sweet cherry leaves to low temperatures under natural conditions 36 d after full bloom (DAFB) was studied by measuring chlorophyll fluorescence parameters, gas exchange parameters, and analyzing biochemical parameters such as chloroplast pigments in leaves. Exposure to low temperatures resulted in changes in all physiological and biochemical parameters studied. There was a uniform and significant decrease in gas exchange and chlorophyll fluorescence parameters and an increase in the content of xanthophyll cycle pigments, especially zeaxanthin and antheraxanthin, and in the de-epoxidation state of the xanthophyll cycle pool.

When sweet cherry trees were exposed to low temperatures, both maximum and actual quantum efficiencies of PS II, net photosynthesis (P_N_), stomatal conductance (*g_s_*), transpiration (Tr), and leaf intercellular CO_2_ concentration (C*_i_*) decreased. The decline was particularly pronounced after the first cold night, when the average 8-h temperature dropped to its lowest value, with a longer duration of minimum temperature and a longer period of temperatures below the threshold of 4.5 °C [21]. Our results suggest that there appears to be no effect of “natural” or “artificial” [19] exposure to low temperatures. Sweet cherry leaves responded in the same way, although the temperature drop was different. In the “artificial” exposure, temperature dropped rapidly to the lowest value and remained about the same during the night, whereas in the “natural” exposure it dropped slowly during the night and reached the lowest value in the early morning.

The decrease in net photosynthesis was associated with stomatal limitations—as shown by the decrease in *g_s_* and C*_i_*. Several studies have reported negative effects of low temperatures on the photosynthetic apparatus in various cold-sensitive plant species [9,10,22,23,24]. Significant reductions in net photosynthesis have also been observed in some fruit species, for example in leaves of two-week-old mulberry seedlings exposed to 3 °C [14], and in leaves of three-year-old sweet cherry trees exposed to 2.2 °C for up to three consecutive nights [19].

Exposure of sweet cherry trees to low temperatures affected thylakoid functions. Some results of chlorophyll fluorescence analysis can be interpreted in terms of photosynthetic activity and provide information about the state of the photosynthetic apparatus and, in particular, the photosystem II (PS II) [25,26]. The decrease in the effective and maximum quantum efficiency of PS II after the first and second nights of cold suggests that the low temperatures most likely caused inactivation of the reaction center PS II or its damage. The results of the present study are consistent with our previous study [19]. A decrease in chlorophyll fluorescence was also observed in leaves of mulberry seedlings exposed at 3 °C [14] and in leaves of grapevine exposed at 5 °C [15,16]. Another possible reason for the cold stress induced decrease in chlorophyll fluorescence in sweet cherry leaves can be attributed to decreased photosynthetic electron transport capacity in PS II [25], structural changes in the PS II complex, changes in non-photochemical quenching (NPQ) efficiency [26], etc.

The reduction in Fv’/Fm’ was probably the result of decreased light utilization due to non-photochemical quenching induced by the xanthophyll cycle [27,28,29], which was also confirmed by biochemical analyses. In the present study and a previous study [20], the de-epoxidation state of xanthophyll cycle pigments (AZ/VAZ) in leaves increased after exposure to low temperatures. The ratio AZ/VAZ expresses the ability to thermally dissipate absorbed excitation energy that accumulates under stress and cannot be used in photosynthesis or dissipated through alternative pathways [28,30]. Exposure to low temperatures increased the capacity for thermal dissipation by accelerating the de-epoxidation state of the xanthophyll cycle and regulating xanthophyll components, thereby protecting the reaction centers of PS II from further damage by excess excitation energy. It has been proposed that the xanthophyll cycle-dependent NPQ is the major mechanism for thermal dissipation of excess energy in higher plants [27,31]. The xanthophyll cycle protects plant metabolism by converting excitation energy into heat, and preventing the formation of ROS, which can damage the photosynthetic apparatus and chloroplasts [30,31]. Regulation of PS II efficiency by NPQ can significantly contribute to reducing the excitation energy reaching the reaction centers of PS II [32,33,34].

Despite the increase in zeaxanthin, a decrease in chlorophylls was observed. Other leaf antioxidants also play a role in protecting chlorophylls from decay, not just carotenoids. In addition, a decrease in violaxanthin was observed, with some violaxanthin being enzymatically converted to antheraxanthin and zeaxanthin as part of the deoxidation process. The increase in antheraxanthin and zeaxanthin was also due to de novo synthesis, which was also observed in 3-week-old maize under cold stress (at 15 °C for 3 days) [35].

The content of chlorophylls and carotenoids changed after the first and second cold night. After the first exposure, the chlorophyll *a*/*b* ratio increased, indicating that cold stress had a greater effect on reducing chlorophyll *b*, as chlorophyll *a* content remained unchanged. Considering that chlorophyll *b* is the second most important photosynthetic pigment next to chlorophyll *a*, this reduction in the ratio could probably be one of the main reasons for the reduced photosynthesis under cold stress. Fernández-Marín, et al. [32] also found that the ratio of chlorophyll *a*/*b* may be increased under stress conditions. Chlorophyll *b* is an accessory pigment in the light-harvesting complex (LHC) and its depletion could be useful in situations where the photosystem cannot convert all the light energy into chemical energy due to stress. In our previous study [20], low temperatures had no effect on leaf chlorophyll, but some other authors reported a decrease [15]. The present results also show lower levels of *β*-carotene and lutein, indicating some damage to the photosystems. *β*-carotene is the only carotenoid in LHC and with lutein in the reaction center of the photosystem II [8].

Both gas exchange and chlorophyll fluorescence parameters recovered within 24 h after one night of cold exposure but not after a second. This suggests that the PS II reaction center of CT2 trees was likely more damaged and therefore recovery time was prolonged. Other studies also suggest that recovery time may be longer with prolonged exposure to low temperatures [9,36,37]. Ibrahim and Bafeel [36] reported that Fv/Fm of *Medicago sativa* leaves recovered within 24 h when exposed to 10 °C for 24 h, Lee and Oh [37] found that Fv/Fm of seedlings of *Brassica oleracea* var. *acephala* exposed to 4 °C for 3 days recovered after 30 h, while Liu, et al. [9] reported an irreversible reduction in photosynthetic rate in tomato seedlings exposed to 6 °C overnight for 9 days.

Chlorophylls, zeaxanthin, and total xanthophylls reached levels similar to controls within 24 h after the first and second nights and antheraxanthin within 48 h. The recovery dynamics of zeaxanthin after exposure to low temperatures in the present study differed from [20] where zeaxanthin was completely epoxidized within 48 h after the second exposure. This might be related to the lower average temperature (1.9 ± 0.1 °C). Overall, our study showed that the recovery of sweet cherry trees exposed to low temperatures probably depends on the duration of exposure and temperature. Most likely, the effects of cold stress were more pronounced when sweet cherry trees were exposed to low temperatures twice, while antioxidants in the leaves were effective enough to protect chlorophylls from further deterioration.

The results presented here show an effect of exposure to low temperatures without frost under natural conditions 36 DAFB on the physiology of sweet cherry leaves. It appears that “natural” exposure to low temperatures elicits a similar response in sweet cherry leaves as “artificial” exposure. Since the dynamics of temperature decline under natural conditions are usually gradual and slow, it was expected that the effect of exposure to low temperatures would not be as pronounced as it actually was.

## 4. Materials and Methods

### 4.1. Plant Material and Experimental Design

**Figure 4:**
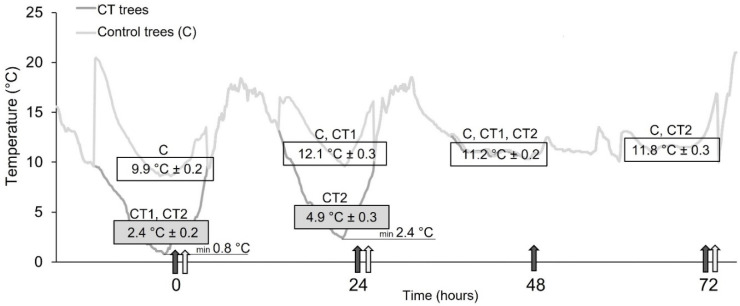
Air temperature (°C) to which trees were exposed on cold nights under natural conditions and in the greenhouse (CT, cold treatment trees; C, control trees), with the time frame (hours after first exposure) of measurements and sampling. The rectangles show the average (±standard error) 8-h temperature (22:00 to 6:00) under natural cold conditions (filled rectangle) and in the greenhouse (empty rectangle). The black arrows indicate measurements of chlorophyll fluorescence parameters and leaf sampling, while the white arrows indicate measurements of gas exchange parameters.

The experiment was conducted on 3-year-old sweet cherry (*Prunus avium* L.) trees at the Biotechnical Faculty of the University of Ljubljana, Slovenia (46°2′ N, 14°28′ E and 297 m a.s.l.). Eighteen trees of the cultivar ‘Grace Star’ on the rootstock ‘Gisela 5′ in 76-litre plastic pots were included in the experiment. Nine trees were randomly assigned to cold treatments (CT) and the other half to controls (C). CT trees were exposed to natural low temperatures 36 DAFB from 6 to 9 May 2019. CT1 trees were exposed to low temperatures on one cold night and CT2 trees were exposed to low temperatures on two cold nights. On the first cold night, all trees from CT were exposed to low temperatures. On the second night, 6 trees were exposed, while the CT1 trees were moved to the greenhouse where they remained all night (Figure 4). Control trees were housed in the greenhouse during the nights and were brought out at sunrise (Figure 4). During the day, all trees were exposed to the same environmental conditions.

The air temperature (°C) during the experiment is shown in Figure 4. On the evening of the first cold night, the air temperature under natural conditions was 9.1 °C and on the second evening at 8 pm it was 12.9 °C. The control trees were moved into the greenhouse at this time, where the temperature was 20.4 °C and 16.5 °C, respectively. The average (±SE) 8-h air temperature (from 22:00 to 6:00 solar time) under natural conditions was 2.4 ± 0.2 °C on the first night and 4.9 ± 0.3 °C on the second night. Temperatures remained below 4.5 °C for 9 h on the first night and for 4 h on the second cold night (threshold for sweet cherry: Zhang, et al. [21]). On the first cold night, temperatures dropped to 0.8 °C for 20 min, but on the second cold night, temperatures dropped to 2.4 °C for 10 min. The average (±SE) 8-h night air temperature in the greenhouse was higher. It was 9.9 ± 0.2 °C on the first night and 12.1 ± 0.3 °C on the second night. On the first cold night, the temperature in the greenhouse dropped to 8.6 °C, but on the second cold night, the temperature dropped to 9.6 °C. The temperature in the greenhouse was above the threshold all night.

### 4.2. Physiological Parameters

#### 4.2.1. Measurements

For physiological measurements, three fully developed, undamaged, and unshaded leaves were marked in the center of each lower branch of each tree. Measurements of chlorophyll fluorescence parameters (effective quantum efficiency of PS II, maximum quantum efficiency of PS II, and electron transport rate) were made at 6:00 a.m. solar time on three marked leaves. Measurements of gas exchange parameters (net photosynthesis, stomatal conductance, transpiration, and leaf intercellular CO_2_ concentration) were made at 10:00 am on one randomly selected leaf from three labelled leaves. All measurements were performed on the same leaf. To obtain a recovery response, physiological measurements were performed several times after exposure to low temperatures. Chlorophyll fluorescence measurements and leaf samples were taken at time 0 (in the morning, when trees were first exposed to low temperatures), and repeated 24, and 48 h later (at time 0, 24, and 48) in CT1 trees and at time 24, 48, and 72 in CT2 trees. Leaf gas exchange parameters were measured 0 and 24 h after the first exposure (at time 0 and 24), and at 0 and 48 h after the second exposure (at time 24 and 72) (Figure 4). Measurements and sampling of controls were performed at the same time.

#### 4.2.2. Chlorophyll Fluorescence Parameters

The maximum quantum efficiency of PS II (Fv/Fm), the effective quantum efficiency of the PS II (Fv’/Fm’), and the electron transport rate (ETR) of PS II were measured with a portable pulse amplitude modulated chlorophyll fluorometer (PAM 2500; Walz, Effeltrich, Germany). For measurements of Fv/Fm, samples were first acclimated to dark conditions for 30 min with special dark adaptation clamps to ensure that all reaction centers were in the open state. Fluorescence was excited with a saturating irradiance of a ‘white light’ pulse (photosynthetic photon flux density, 8000 μmol m^−2^s^−1^; 0.8 s).

#### 4.2.3. Gas Exchange Parameters

Measurements were performed using a portable photosynthesis system LI6400xt (LI-COR, Lincoln, NE, USA) with IRGA detector. Net photosynthesis (P_N_), stomatal conductance (*g_s_*), transpiration (Tr), and leaf intercellular CO_2_ concentration (C*_i_*) were recorded at constant reference CO_2_ concentration (400 μmol mol^−1^), saturating light intensity (photosynthetic photon flux density = 1000 μmol m^−2^s^−1^), and relative humidity in the chamber (approximately 70%).

### 4.3. Biochemical Parameters

#### 4.3.1. Samplings and Sample Preparation

For chloroplast pigment analysis, five fully developed and undamaged leaves with similar exposure were randomly sampled from each tree before sunrise (at 6:00 a.m. solar time) at time 0 for CT1 trees and at time 24 for CT2 trees. Sampling was repeated after 24 and 48 h (at time points 24 and 48) for CT1 trees and 48 and 72 for CT2 trees (Figure 4) to obtain a recovery response. Until further analysis, sampled leaves were frozen in liquid nitrogen, lyophilized, ground to a fine powder, and stored in moisture-proof, dark plastic containers at −20 °C.

#### 4.3.2. Extraction and HPLC Analysis of Chloroplast Pigments

Lyophilized dry leaf powder (0.1 g) was homogenized with 100% cold acetone (3 mL) using Ultra-Turrax homogenizer for 30 s. Samples were then centrifuged at 11,500× *g* and 4 °C for 10 min, filtered through Minisart (SRP 15, PTFE) polyamide filters (Sartorius Stedim Biotech, Göttingen, Germany) and transferred to vials. The entire extraction process was performed in dim light.

Chloroplast pigments were analyzed using HPLC-DAD (Thermo Finnigan, San Jose, CA, USA) according to the method described in Vosnjak, et al. [20]. The content of each compound was calculated using corresponding external standards and expressed as µg g^−1^ dry weight (DW).

The following sums of compounds identified in our study were expressed as total chlorophylls (sum of chlorophyll *a* and chlorophyll *b*), total carotenoids (sum of lutein, *β*-carotene, neoxanthin, violaxanthin (V), antheraxanthin (A) and zeaxanthin (Z)), xanthophyll cycle pigments (VAZ; sum of V, A and Z). In addition, de-epoxidation state of the xanthophyll cycle (AZ/VAZ; ratio between the sum of A and Z and the sum of V, A and Z) and the chlorophyll *a*/*b* ratio (ratio between chlorophyll *a* and chlorophyll *b*) were calculated.

### 4.4. Statistical Analysis

Statistical analysis was performed using R statistical software version 3.6.1 (R foundation for Statistical Computing, Vienna, Austria) [22]. The experiment was a factorial design, and the data were analyzed as a single—factorial experiment, combining two factors, “CT treatment” and “time after exposure,” into one factor CT.time. For the chlorophyll fluorescence parameters and chloroplast pigment data, the factor CT.time had 10 levels (CT1.0, CT1.24, CT1.48, CT2.24, CT2.48, CT2.72, C.0, C.24, C.48, C.72) and for the gas exchange parameter data, it had 7 levels (CT1.0, CT1.24, CT2.24, CT2.48, C.0, C.24, C.72). When analysis of variance showed statistical significance, contrast analysis was performed, using the glht function with user-defined contrasts from the R package multcomp. *p* values less than 0.05 were considered statistically significant. Contrasts between the means of the variables were determined according to the structure of the CT.time treatments compared with the control, within the same time.

## 5. Conclusions

One- or two-night exposures of 3-year-old sweet cherry trees to low temperatures under natural conditions, 36 DAFB significantly altered leaf physiological and biochemical parameters. Exposure reduced both Fv/Fm and Fv’/Fm’, decreased leaf net photosynthesis, stomatal conductance, chlorophyll *b*, total chlorophyll, and lutein content and increased zeaxanthin and antheraxanthin content. The two cold nights further increased zeaxanthin content. Exposure to low temperatures on two cold nights induced stress, but the leaf response varied with the number of exposures and temperature. Two exposures caused more stress than one exposure and prolonged the recovery time of Fv/Fm and the de-epoxidation state of the xanthophyll cycle pool.

## Figures and Tables

**Table 1:** The results of contrast analysis of physio-biochemical parameters in leaves of sweet cherry trees exposed to one (CT1) or two (CT2) cold nights. Measurements and samplings were performed at time 0, 24, and 48 for CT1 trees and at time 24, 48, and 72 for CT2 trees and compared with the corresponding controls.

	CT1			CT2		
	0	24	48	24	48	72
**Chlorophyll fluorescence parameters**						
Fv/Fm	↓ ***	ns	ns	↓ ***	↓ *	ns
Fv’/Fm’	↓ ***	ns	ns	↓ *	ns	ns
ETR	ns	ns	ns	↑ **	ns	ns
**Gas exchange parameters**						
P_N_	↓ ***	ns	―	ns	―	↓ **
Tr	↓ ***	ns	―	ns	―	ns
*g_s_*	↓ ***	ns	―	ns	―	↓ ***
C*_i_*	↓ ***	ns	―	ns	―	ns
**Biochemical parameters**						
chlorophyll *a*	ns	ns	ns	ns	ns	ns
chlorophyll *b*	↓ *	ns	ns	↓ *	ns	ns
chlorophyll *a*/*b*	↑ *	ns	ns	ns	ns	ns
total chlorophylls	↓ *	ns	ns	↓ *	ns	ns
*β*-carotene	↓ **	ns	ns	↓ *	↓ *	↓ *
lutein	↓ **	↓ ***	ns	↓ *	ns	ns
neoxanthin	ns	ns	ns	ns	ns	ns
violaxanthin	↓ **	ns	ns	ns	ns	↓ *
antheraxanthin	↑ ***	↑ **	ns	↑ **	↑ *	ns
zeaxanthin	↑ ***	ns	ns	↑ ***	ns	ns
total carotenoids	ns	ns	ns	ns	ns	ns
VAZ	↑ ***	ns	ns	↑ ***	ns	ns
AZ/VAZ	↑ ***	↑ **	ns	↑ ***	↑ *	↑ *

*, significant differences at *p* < 0.05; **, significant differences at *p* < 0.01; ***, significant differences at *p* < 0.001; ns, not significant; ↑ = increase regarding to corresponding controls; ↓ = decrease regarding to corresponding controls; ―, not monitored; Fv/Fm, maximum quantum efficiency of PS II; Fv’/Fm’, effective quantum efficiency of PS II; ETR, electron transport rate; P_N_, net photosynthesis; Tr, transpiration; *g_s_*, stomatal conductance; C*_i_*, intercellular leaf CO_2_ concentration; VAZ, xanthophyll cycle pigments; AZ/VAZ, de-epoxidation state of xanthophyll cycle pool.

## Data Availability

Not applicable.
